# The molecular logic of sodium-coupled neurotransmitter transporters

**DOI:** 10.1098/rstb.2008.0181

**Published:** 2008-10-31

**Authors:** Eric Gouaux

**Affiliations:** Howard Hughes Medical Institute, Vollum Institute, Oregon Health and Science UniversityPortland, OR 97239, USA

**Keywords:** sodium-coupled transporters, neurotransmitters, chemical synapses, crystallography

## Abstract

Synaptic transmission at chemical synapses requires the removal of neurotransmitter from extracellular spaces. At synapses in the central nervous system, this is accomplished by sodium-coupled transport proteins, integral membrane proteins that thermodynamically couple the uptake of neurotransmitter to the uptake of sodium and, in some cases, the uptake and export of additional ions. Recent X-ray crystallographic studies have revealed the architecture of the two major families of neurotransmitter transporters and, together with additional biochemical and biophysical studies, have provided insights into mechanisms of ion coupling, substrate uptake, and inhibition of transport.

A fundamental mechanism by which nerve cells communicate with one another involves the stimulated release of a neurotransmitter by one cell and the detection of the neurotransmitter by one or more adjacent cells at specialized junctions called synapses. The basic principle underlying this mode of communication involves the orchestrated release and uptake of neurotransmitter molecules (Lisman *et al*. 2007). At glutamatergic synapses, for example, the basal (resting) concentration of extracellular glutamate is approximately 25 nM (Herman & Jahr 2007), whereas immediately after release the concentration rises about 10^5^-fold to millimolar concentrations (Wadiche & Jahr 2001). This pulse of neurotransmitter activates both ionotropic and metabotropic receptors, resulting in membrane depolarization, calcium influx and activation of intracellular signalling pathways. To extinguish the bolus of extracellular neurotransmitter, synapses in the central nervous systems are surrounded by integral membrane transport proteins that drive the uptake of neurotransmitter into cells by coupling transport to the thermodynamically favourable movement of one or more ions (Tzingounis & Wadiche 2007). In this review I will focus on mechanistic principles of sodium-coupled neurotransmitter transporters revealed from crystallographic and other biophysical studies of prokaryotic orthologues.

There are two families of plasma membrane-localized neurotransmitter transporters, each distinct in amino acid sequence and atomic structure, which carry out the majority of transmitter clearance: transporters for the inhibitory transmitters glycine and γ-amino butyric acid (GABA), and the biogenic amines (dopamine, noradrenaline, serotonin; known as the neurotransmitter sodium symporter (NSS) or the SLC6 family; Chen *et al*. 2004); and transporters for excitatory transmitter glutamate and the neutral amino acids alanine, serine, cysteine and threonine (also known as the excitatory amino acid transporters or EAATs and the ASCT transporters, respectively, both members of the SLC1 family; Kanai & Hediger 2004). Many orthologues of these eukaryotic transport proteins are found in prokaryotes, and over the past several years we have studied LeuT, a eubacterial orthologue of the glycine, GABA and biogenic amine family, and GltPh, an archebacterial orthologue of the aspartate/glutamate and neutral amino acid transporter family (figure 1).

Upon solving the crystal structures of LeuT (Yamashita *et al*. 2005) and GltPh (Yernool *et al*. 2004) we discovered both transporters in a substrate- and ion-bound state in which access to the binding sites, from both sides of the membrane, was blocked by protein residues (figure 2). We have deemed these substrate- and ion-bound states as occluded states. How did we define these states as being ‘occluded’? To do this we used two criteria. First, we displayed the calculated solvent accessible surface of the protein, computed using a probe with 1.4 Å radius, and asked whether or not the substrates or ions were accessible to the probe. In both cases, substrates and ions were largely inaccessible to the probe. Second, we asked whether or not there was an unobstructed pathway large enough to permit passage of substrate from the binding site to bulk solution. Again, for both LeuT and GltPh, there was not a pathway large enough to allow for release of substrate. In other words, release of substrate would require a conformational change of the protein. Thus, LeuT and GltPh have occluded substrate binding sites.

A common observation from both calculations, however, was that the ‘thickness’ of protein occluding the substrate/ion binding pockets from the extracellular side of the membrane was much smaller than that shielding the binding site from the cytoplasmic side. Thus, for both LeuT and GltPh, we suggested that access to the binding sites from the outside would require fewer and perhaps less substantial conformational changes in comparison with the changes that would be required for access to the binding sites from the inside or cytoplasmic solution. Although we have only solved a few structures, and thus our present conclusions must be correspondingly tempered, I nevertheless suggest that the less substantial extracellular barrier or gate might allow for substrates and ions to bind and unbind more rapidly from the extracellular solution in comparison to the intracellular solution, i.e. opening to extracellular solution is kinetically ‘easier’—the barrier is lower. Furthermore, open-to-the-outside states may be thermodynamically more stable than states that are open to the inside, and thus LeuT and GltPh may tend to preferentially occupy open-to-the-outside states, ready for substrate and ion binding. Different transporters, however, are likely to have extracellular and intracellular gates of different ‘strengths’ and thus may favour different conformations. Perturbations of the transporter, by phosphorylation, by interaction with partner proteins or by interactions with small molecules and ions, provide additional mechanisms by which the conformational preference of a specific transporter may be modulated.

In the case of LeuT, the barrier to the outside, or extracellular gate, is formed primarily by Tyr108 (TM3), Phe259 (TM6), Arg30 (TM1) and Asp404 (TM10) (Yamashita *et al*. 2005), whereas in GltPh the extracellular gate is composed of residues at the tip of hairpin 2 (HP2; Yernool *et al*. 2004). Even though there are only several residues ‘covering’ the substrates and ions in the substrate and ion-bound forms of LeuT and GltPh, I suggest that these bound or occluded states of the transporter are substantially different in conformation from the apo or unliganded states. For GltPh, we know that in the apo and dl-threo-β-benzyloxyaspartic acid (TBOA)-inhibited states, HP2 opens and exposes the substrate binding site to the extracellular solution, thus demonstrating, by extension, that upon substrate and ion binding, HP2 closes and occludes substrate and ions in their binding pockets (Boudker *et al*. 2007). These observations thus lead us to a simple mechanistic scheme shown in figure 3.

If the occluded state observed in our crystallographic studies is a *bona fide* intermediate along the transporter reaction pathway, and if we could find a small molecule that either stabilizes the occluded state or raises energetic barriers to its conversion to open-to-the-inside or open-to-the-outside conformations, then the compound should inhibit transporter catalysed substrate uptake. Upon screening a series of inhibitors of eukaryotic NSS transporters, we discovered that tricyclic antidepressants that include clomipramine were inhibitors of LeuT-catalysed ^3^H-Leu and ^3^H-Ala uptake (Singh *et al*. 2007). On the basis of detailed kinetic experiments using LeuT reconstituted into proteoliposomes, we determined that clomipramine inhibited LeuT by way of a non-competitive mechanism. High-resolution crystallographic studies of LeuT in complex with multiple tricyclic antidepressants (TCAs) showed that one TCA molecule bound at the base of the extracellular vestibule, further occluding leucine and the two sodium ions (figure 4). The location of the TCA molecule suggests that it inhibits transport by either stabilizing the occluded state or by raising the energetic barrier for opening to the inside or to the extracellular solution (Singh *et al*. 2007). To test the hypothesis that TCAs stabilize the occluded state, we carried out off-rate studies using ^3^H-Leu bound LeuT. In accord with our predictions, we found that a saturating concentration of clomipramine slowed the rate at which ^3^H-Leu falls off LeuT by approximately 700-fold (Singh *et al*. 2007), thus providing experimental justification for the mechanism by which TCAs inhibit LeuT (figure 5).

Related studies by Wang, Reith and colleagues on LeuT and TCAs appeared at about the same time as our studies (Zhou 2007). A very important difference between the conclusions drawn from the two studies is that Wang, Reith and colleagues argued that the TCA site in LeuT is directly equivalent to the TCA site in the human serotonin transporter (hSERT). We do not believe this is the case for two important reasons. First, the kinetic mechanism of TCA inhibition of the eukaryotic serotonin transporter is competitive (Talvenheimo *et al*. 1979), whereas that for LeuT is non-competitive (Singh *et al*. 2007). Second, amino acid residues that profoundly perturb TCA binding in the eukaryotic SERT are located at the substrate binding site (Henry 2006) and not at the TCA vestibule binding site defined by the LeuT complexes. Therefore, we do not believe the LeuT TCA site identified at the base of the extracellular vestibule is equivalent to the TCA site in hSERT. Furthermore, there are a number of additional recent studies (Henry 2006; Paczkowski *et al*. 2007; Beuming 2008; Parnas 2008) that strongly disfavour the conclusion of Wang, Reith and colleagues.

The first crystal structure of GltPh was solved at the modest resolution of approximately 3.5 Å and although we found non-protein electron density situated between the tips of HP1 and HP2, we were not able to conclusively determine that the density was associated with a bound substrate (Yernool *et al*. 2004; figure 6*a*). Subsequent studies at higher resolution (3.0–3.2 Å), together with careful anomalous diffraction measurements from crystals formed with the substrate analogue cysteine sulfinic acid, conclusively demonstrated that the non-protein density was unambiguously due to bound substrate (Boudker *et al*. 2007). Fitting of substrate (aspartate) into this electron density demonstrated that the substrate formed multiple interactions with HP1, HP2, TM7 and TM8, probably stabilizing HP2 in a closed conformation (figure 6*b*; Boudker *et al*. 2007).

Determination of aspartate as the ligand bound to GltPh and as a transport competent substrate led us to ask whether the broad spectrum competitive inhibitor of eukaryotic transporters TBOA (Shimamoto 1998), might also act as a competitive inhibitor of GltPh. On the basis of ^3^H-Asp uptake experiments using purified GltPh reconstituted into proteoliposomes, we showed that TBOA does in fact inhibit transport (Boudker *et al*. 2007). These results then prompted us to determine the structural basis for TBOA inhibition.

We solved the structure of TBOA bound to GltPh and found that in the complex with TBOA, HP2 adopts an open conformation, with residues at the tip displaced as much as 10 Å from the closed, occluded state conformation (Boudker *et al*. 2007; figure 7). This conformational change is the simple consequence of the bulky benzyl group on TBOA; the aspartate moiety of TBOA binds in the same pocket as the substrate aspartate and the benzyl group is lodged against HP2, propping it in an open conformation and preventing HP2 closure. The open conformation of HP2 has the added consequence of disrupting sodium site 2 and, as one would predict from the structure, the sodium dependence of TBOA binding is approximately 1, substantially less than the sodium dependence of aspartate binding, which is approximately 2.

With crystal structures of GltPh in multiple states, we can therefore construct a speculative reaction scheme for ion-driven substrate transport and for competitive inhibition by TBOA. As illustrated in figure 8, we suggest that in the apo state, GltPh is predominantly in a conformation in which HP2 adopts an open conformation, exposing the substrate and ion-binding sites to the extracellular solution. Upon sodium and substrate binding, HP2 closes and the transporter forms the occluded state like that seen in the initial GltPh crystal structure. Once in the occluded state, one of two events can occur: HP2 can reopen and substrates and ions can unbind to the extracellular solution, or HP1, together with other, yet to be identified elements of the cytoplasmic gate, undergo a conformational change allowing substrates and ions access to the cytoplasmic solution. Because the concentration of sodium is low in the cytoplasm, and the binding of substrate and sodium is tightly coupled (Boudker *et al*. 2007), rebinding of substrate and sodium to the transporter, from the intracellular solution, is thermodynamically unfavourable. Thus, sodium-driven substrate transport is the consequence of tightly coupled substrate and sodium binding, formation of the occluded state and unbinding of substrate and sodium ions into the sodium-depleted intracellular solution. As illustrated in figure 8*d*, competitive inhibition of GltPh-catalysed aspartate uptake is the simple consequence of stabilization of HP2 in an open-to-the-outside conformation, a conformation that not only precludes the closure of HP2 and formation of the occluded state but also prevents the binding of the crucial second sodium ion.

In conclusion, the general principles of non-competitive and competitive inhibition seen with LeuT and GltPh that involve stabilization of an occluded state and an open-to-the-outside conformation, respectively, may be broadly applicable to other transport proteins, even though the specific molecular structures and details will certainly be different.

## Figures and Tables

**Figure 1:**
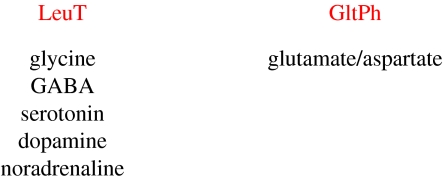
LeuT and GltPh are bacterial orthologues of the two major families of the neurotransmitter transporters.

**Figure 2:**
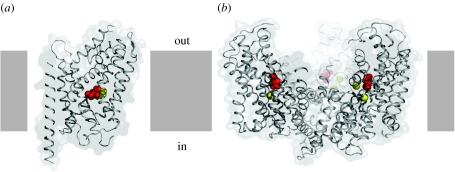
The first crystal structures of (*a*) LeuT (Yamashita *et al*. 2005) and (*b*) GltPh (Yernool *et al*. 2004; Boudker *et al*. 2007) revealed substrate (red) and ions (yellow) bound close in space, about halfway across the membrane bilayer, within occluded sites shielded by protein from solution on both the extracellular and intracellular sides of the membrane.

**Figure 3:**
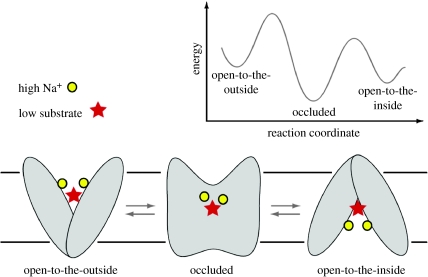
A mechanism to describe the activity of LeuT and GltPh that involves binding of substrates and ions to an open-to-the-outside conformation, isomerization to an occluded state, and subsequent isomerization to an open-to-the-inside conformation.

**Figure 4:**
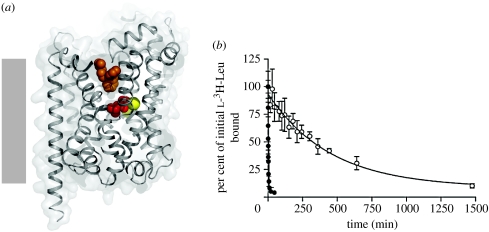
Complex of LeuT with imipramine (Singh *et al*. 2007). (*a*) Tricyclic antidepressants, such as imipramine (orange) bind in the extracellular vestibule of LeuT, above the occluded leucine (red) and sodium ions (yellow) (Singh *et al*. 2007). (*b*) Clomipramine slows the off-rate of ^3^H-leucine by approximately 700-fold. Off-rate measurements carried out in the absence (filled circles) and presence (open circles) of 3 mM clomipramine (Singh *et al*. 2007).

**Figure 5:**
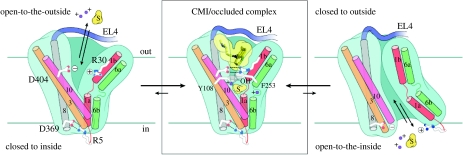
Mechanism by which tricyclic antidepressants inhibit the activity of LeuT.

**Figure 6:**
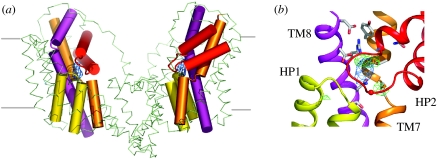
GltPh has an occluded substrate binding site. (*a*) Electron density for substrate situated in a pocket underneath hairpin 2 (HP2; red), above HP1 (yellow), and adjacent to TM7 (orange) and TM8 (purple) (Yernool *et al*. 2004). (*b*) Close-up of substrate electron density (blue mesh) and sulphur anomalous density associated with bound cysteine sulfinic acid (green mesh) and interacting residues on HP1, HP2, TM7 and TM8. Colouring is the same as in (*a*) (Boudker *et al*. 2007).

**Figure 7:**
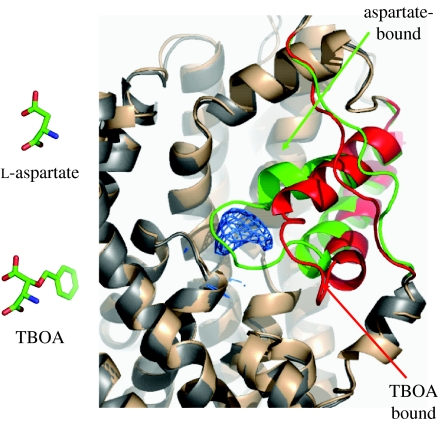
The competitive inhibitor TBOA stabilizes HP2 in an outward-facing conformation, thus blocking the binding of a second sodium ion, closure of HP2 and opening of the transporter to the inside (Boudker *et al*. 2007).

**Figure 8:**
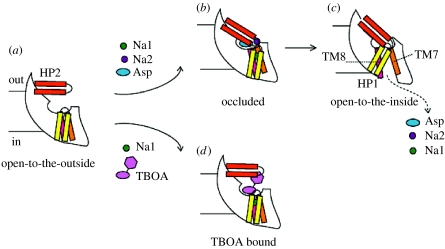
(*a–d*) A mechanism to describe the activity of GltPh and its inhibition by competitive inhibitors such as TBOA (Boudker *et al*. 2007).
